# Functional redundancy buffers the effect of poly-extreme environmental conditions on southern African dryland soil microbial communities

**DOI:** 10.1093/femsec/fiae157

**Published:** 2024-11-20

**Authors:** Tomás Sauma-Sánchez, Jaime Alcorta, Javier Tamayo-Leiva, Beatriz Díez, Hugo Bezuidenhout, Don A Cowan, Jean-Baptiste Ramond

**Affiliations:** Extreme Ecosystem Microbiomics & Ecogenomics (E²ME) Laboratory, Facultad de Ciencias Biológicas, Pontificia Universidad Católica de Chile, Santiago 8331150, Chile; Microbial Ecology of Extreme Systems Laboratory, Facultad de Ciencias Biológicas, Pontificia Universidad Católica de Chile, Santiago 8331150, Chile; Millennium Institute Center for Genome Regulation (CGR), Santiago 8331150, Chile; Microbial Ecology of Extreme Systems Laboratory, Facultad de Ciencias Biológicas, Pontificia Universidad Católica de Chile, Santiago 8331150, Chile; Millennium Institute Center for Genome Regulation (CGR), Santiago 8331150, Chile; Center for Climate and Resilience Research (CR)^2^, Santiago 8370449, Chile; Microbial Ecology of Extreme Systems Laboratory, Facultad de Ciencias Biológicas, Pontificia Universidad Católica de Chile, Santiago 8331150, Chile; Millennium Institute Center for Genome Regulation (CGR), Santiago 8331150, Chile; Center for Climate and Resilience Research (CR)^2^, Santiago 8370449, Chile; Scientific Services Kimberley, South African National Parks, Kimberley 8306, South Africa; Applied Behavioural Ecology & Ecosystem Research Unit, UNISA, P/Bag X6, Florida 1710, South Africa; Centre for Microbial Ecology and Genomics, Department of Biochemistry, Genetics and Microbiology, University of Pretoria, P/Bag X20, Pretoria 0028, South Africa; Extreme Ecosystem Microbiomics & Ecogenomics (E²ME) Laboratory, Facultad de Ciencias Biológicas, Pontificia Universidad Católica de Chile, Santiago 8331150, Chile; Centre for Microbial Ecology and Genomics, Department of Biochemistry, Genetics and Microbiology, University of Pretoria, P/Bag X20, Pretoria 0028, South Africa

**Keywords:** dryland soils, edaphic microbial communities, functional redundancy, metabarcoding, niche partitioning, shotgun metagenomics

## Abstract

Drylands’ poly-extreme conditions limit edaphic microbial diversity and functionality. Furthermore, climate change exacerbates soil desiccation and salinity in most drylands. To better understand the potential effects of these changes on dryland microbial communities, we evaluated their taxonomic and functional diversities in two Southern African dryland soils with contrasting aridity and salinity. Fungal community structure was significantly influenced by aridity and salinity, while Bacteria and Archaea only by salinity. Deterministic homogeneous selection was significantly more important for bacterial and archaeal communities’ assembly in hyperarid and saline soils when compared to those from arid soils. This suggests that niche partitioning drives bacterial and archaeal communities' assembly under the most extreme conditions. Conversely, stochastic dispersal limitations drove the assembly of fungal communities. Hyperarid and saline soil communities exhibited similar potential functional capacities, demonstrating a disconnect between microbial structure and function. Structure variations could be functionally compensated by different taxa with similar functions, as implied by the high levels of functional redundancy. Consequently, while environmental selective pressures shape the dryland microbial community assembly and structures, they do not influence their potential functionality. This suggests that they are functionally stable and that they could be functional even under harsher conditions, such as those expected with climate change.

## Teaser

Salinity and aridity shape the assembly and structures, but not the potential functionality, of microbial communities from Southern African dryland soils.

## Introduction

Drylands are defined by having an aridity index, the ratio of the mean annual precipitation to potential evapotranspiration over a multi-annual period, <0.65 (United Nations Convention to Combat Desertification 2017). They are present in every continent and cover 45.4% of Earth's terrestrial surface (66.7 Mkm^2^) (Prăvălie 2016). Globally, 74% of all pastures and 50% of all croplands are located in drylands, which are inhabited by a third of the global human population (>2.8 billion people) (Plaza et al. 2018). Although it is not clear if the trends of current climate change are globally increasing the total dryland surface (Huang et al. 2016, Berg and McColl 2021), there is high confidence that climate change is exacerbating the vulnerability of these habitats to desertification (i.e. land degradation in dry sub-humid, semi-arid, and arid drylands) (Mirzabaev et al. 2019). This phenomenon intensifies the processes of soil salinization and aridification (Mirzabaev et al. 2019), which may have deleterious effects on these highly vulnerable ecosystems (e.g. by significantly decreasing net primary production) and their human populations (e.g. reducing food security) (United Nations Convention to Combat Desertification 2017).

Drylands are also characterized by soils with high salinity and limited nutrient availability, diurnal and seasonal thermal extremes, and high ultraviolet irradiation (Makhalanyane et al. 2015). As a result, drylands have low annual litter decomposition, nutrient cycling, respiration, and primary productivity rates (Talmon et al. 2011, Pointing and Belnap 2012, Cordero et al. 2018, Plaza et al. 2018, Liu et al. 2019). Since the presence of plants and animals is generally limited by these poly-extreme conditions, soil microbial communities are crucial for ecosystem functional processes, particularly for nutrient cycling and above-ground net primary production (Maestre et al. 2016, Cowan et al. 2020, Hu et al. 2021, Ramond et al. 2022). With high levels of genomic plasticity and metabolic capacity, microorganisms have developed numerous strategies to resist, tolerate and even thrive in drylands (e.g. Rao et al. 2016, Jordaan et al. 2020, Leung et al. 2020, Meier et al. 2021). Microbial populations particularly colonize refugee niches (e.g. hypolithic, endolithic, and biological soil crust communities) (Pointing and Belnap 2012), but are also found active in open superficial and sub-superficial soils (Gunnigle et al. 2014, 2017, Schulze-Makuch et al. 2018, León-Sobrino et al. 2019, Cowan et al. 2020).

Dryland edaphic microbial communities have been extensively studied worldwide (e.g. Pointing and Belnap 2012, Makhalanyane et al. 2015, Neilson et al. 2017, León-Sobrino et al. 2019, Bay et al. 2021, Li et al. 2022, Ramond et al. 2022), revealing numerous patterns in community structure and functionality across climatic and environmental gradients. Increasing aridity leads to decreasing levels of soil total nitrogen and organic carbon availability (Delgado-Baquerizo et al. 2013a,b), microbial biomass and diversity (Maestre et al. 2015), and soil multifunctionality (Hu et al. 2021). Furthermore, higher aridity increases the relative abundance of stress response genes while decreasing that of nutrient cycling genes (Fierer et al. 2012, Song et al. 2019, Li et al. 2022). These patterns have been reported in Southern African soils where mean annual precipitation was negatively correlated with soil vegetation coverage and nutrient content [i.e. carbon content (C), phosphorous (P), and ammonium (NH_4_^+^)], and indirectly correlated, through its effects on the soil chemistry, both positively and negatively, with microbial diversity and functionality (Cowan et al. 2022, Naidoo et al. 2022, Vikram et al. 2023). Similarly, salinity has been identified as one of the most important factors influencing dryland soil microbiomes: reducing community diversity, increasing the relative importance of deterministic processes such as environmental filtering, and substantially changing the composition and functional capacities of edaphic communities (Magalhães et al. 2014, Johnson et al. 2017, Ren et al. 2018, Scola et al. 2018, Zhang et al. 2019). However, the impact of these environmental conditions on microbial communities is not consistent for Bacteria, Archaea, and Fungi. Due to differences in their growth habits, physiology, and dispersal mechanisms, fungal communities tend to assemble in a highly stochastic manner, while bacterial and archaeal communities rather by deterministic mechanisms (Powell et al. 2015, Vikram et al. 2023).

Each dryland, including their different soil biotopes such as gravel plains, sand dunes, river beds, salt pans, and playas, presents distinct microbial assemblages (Crits-Christoph et al. 2013, Gombeer et al. 2015, Makhalanyane et al. 2015, Ronca et al. 2015, van der Walt et al. 2016, Johnson et al. 2017, Schulze-Makuch et al. 2018, Li et al. 2023). The extensive heterogeneity of dryland ecosystems makes it essential that both large- and small-scale surveys are used to fully understand the scope and complexity of dryland soil microbiomes. We, therefore, evaluated how edaphic microbial communities assemble and potentially function under contrasted poly-extreme conditions (arid vs. hyperarid/saline vs. non-saline) in Southern African drylands using a metabarcoding and shotgun metagenomics approach. We hypothesized that the structure of the edaphic microbial communities would differ in each environmental setting studied (i.e. between drylands), but also between more closely located sites (>5 km) within each dryland, suggesting that environmental filtering would be a dominant driver of community structure and functionality in such poly-extreme environments (Ronca et al. 2015, Johnson et al. 2017, Scola et al. 2018, Cowan et al. 2022). Also, we expect Bacteria and Archaea communities to be more deterministically assembled than fungal communities which, notably due to dispersal limitation mechanisms, are likely to exhibit a more unpredictable assembly (Powell et al. 2015, Vikram et al. 2023, Powell and Bennett 2016). We further predicted that the harshest edaphic environment (i.e. the hyperarid and saline soils), would harbor a less functionally diverse communities, but that it would be enriched in stress-related genes (Fierer et al. 2012, Song et al. 2019, Li et al. 2022).

## Materials and methods

### Field sampling and physicochemical characterization

**Figure 1.**
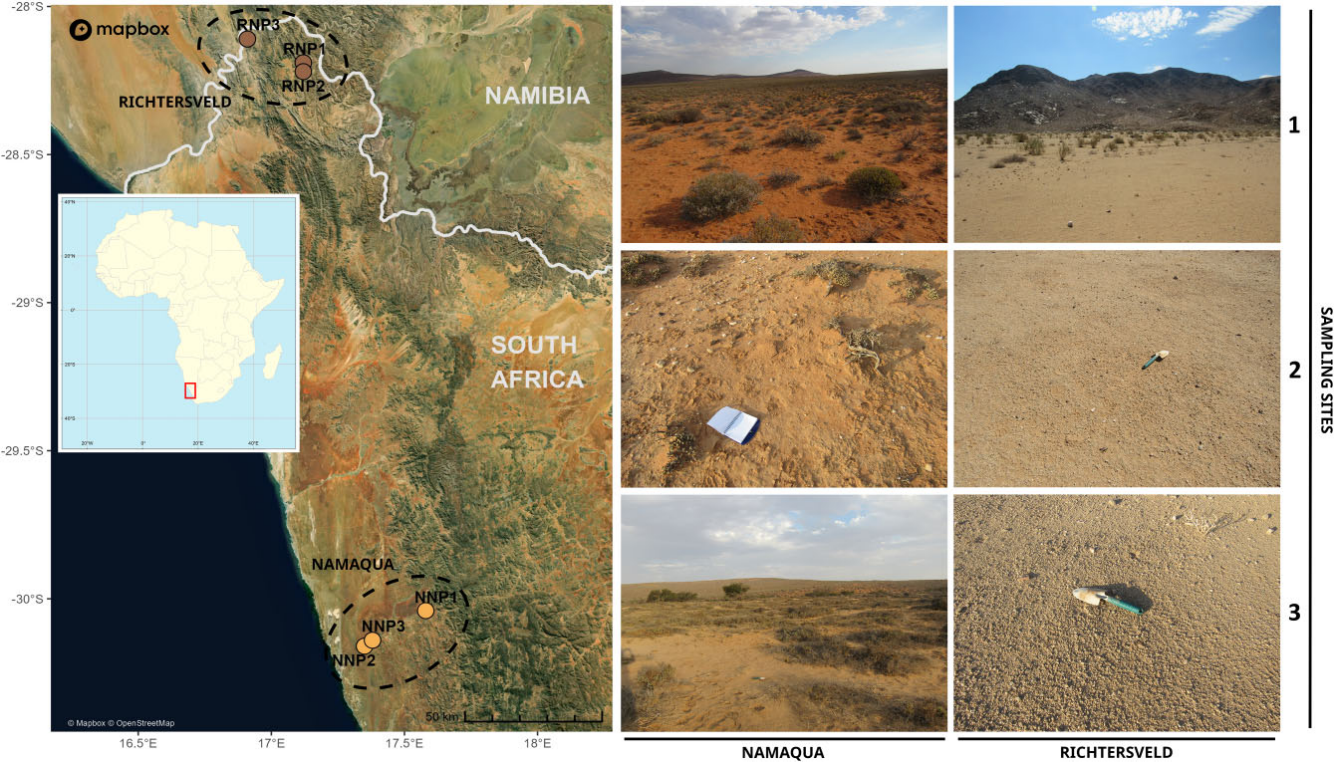
Map of South Africa with the sampling sites. The map shows the distribution of the sampling sites colored according to their dryland of origin (Namaqua and Richtersveld). Representative photographs of each sample site are included (photos courtesy of *J.-B. Ramond*). Satellite images were produced using © Mapbox.

Field sampling took place in March 2017 in the arid Namaqua National Park (NNP) and hyperarid Richtersveld National Park (RNP), under the authority of South African National Parks (SANParks), with sampling permit reference RAMJ1384 (Fig. 1). Surface soil samples (0–2 cm) were collected at three individual and distant (>5 km) sites. At each site, four true replicates (located ∼50 m apart) of plant-free soil were collected (*n* = 12 per dryland) using sterile methods. Samples for soil physicochemistry analyses were stored at room temperature, while the samples for molecular analyses were stored at –20⁰C.

A total of 14 physicochemical properties were measured for each soil sample (Table S1). Soil potassium (K^+^), calcium (Ca^2+^), magnesium (Mg^2+^), sodium (Na^+^), chloride (Cl^−^), ammonium (NH_4_^+^), nitrate (NO_3_^−^), and phosphorous (P) contents, electric conductivity (EC), and pH were determined by Bemlabs (Pty) Ltd. (Strand, Western Cape, South Africa). Total carbon percentage (%C) was determined using the Walkley–Black chromic acid wet oxidation method (Walkley 1935), and soil texture (percentages of sand, silt, and clay) using the hydrometer method (Bouyoucos 1962) at the Soil Science Laboratory of the University of Pretoria, South Africa.

### Metagenomic DNA extraction and sequencing

Metagenomic DNA (mDNA) was extracted from 0.5 g of soil using the PowerSoil® DNA Isolation Kit (MO BIO, West Carlsbad, CA) according to the manufacturer's instructions. The extracted mDNA was quality checked on 1% agarose gels. The concentration and purity of the mDNA were determined using a Nanodrop 2000 UV-Vis spectrophotometer (Thermo Scientific).

The extracted mDNA was sent for amplicon (*n* = 24; 12 per dryland) and shotgun (*n* = 2; 1 per dryland) sequencing to MrDNA (Shallowater, TX, USA). The Illumina MiSeq sequencing platform with paired-end technology (2×150 bp) was used for amplicon sequencing. The bacterial and archaeal 16S rRNA gene V4 variable region (255 bp) was amplified using the Reagent Kit V3, and the forward 515F (5′-GTGCCAGCMGCCGCGGTAA-3′) and reverse 806R (5′-GGACTACNVGGGTWTCTAAT-3′) primer set (Varliero et al. 2023), producing paired-end reads (300 bp). The eukaryotic nuclear ribosomal internal transcribed spacer (ITS) region was amplified using the forward ITS1 (5′-CTTGGTCATTTAGAGGAAGTAA-3′) and reverse ITS2 (5′-GCTGCGTTCTTCATCGATGC-3′) primer set (Anderson and Cairney 2004).

Prior to shotgun sequencing, mDNA extracted from the four true-replicate soils from a single site in each dryland (site 3) were pooled together in equal proportions based on their molecular weight and DNA concentrations. Subsequently, libraries were prepared using a Nextera DNA Sample Preparation Kit (Illumina Inc., San Diego, CA, USA) according to the manufacturer instructions, and sequenced using the Illumina HiSeq2000 (2×250 bp), yielding a total of 113 981 269 and 120 467 332 paired reads for Namaqua and Richtersveld samples, respectively.

### Amplicon reads pre-processing

Raw amplicon reads were processed with QIIME 2 v2022.2 (Bolyen et al. 2019). Demultiplexed reads with a Standard Phred score (Q) <30 were removed. Adaptors were removed from the reads, and a minimum length of 200 bp was defined using Cutadapt QIIME 2 plugin (Martin 2011). Denoising and amplicon sequence variants (ASVs) inference were performed for both 16S rRNA and ITS data sets using the Deblur pipeline (Amir et al. 2017), obtaining a total of 7867 and 755 ASVs from the 16S and ITS datasets, respectively. A median of 42 777 reads and 1 609 ASVs were detected per sample for the 16S marker, while 16 557 reads and 143 ASVs were detected per sample for the ITS. For the taxonomic classification of the ASVs, naïve Bayes classifiers were trained using the 16S rRNA reference sequences extracted from the SILVA r138 SSURef NR99 dataset (Quast et al. 2013), and the full reference sequences from the UNITE v8.3 database for fungi (Nilsson et al. 2019, Kõljalg et al. 2020). Finally, an approximately-maximum-likelihood rooted phylogenetic tree was generated for each data set using Multiple Alignment using Fast Fourier Transform (MAFFT) alignment and FastTree pipeline of the phylogeny QIIME 2 plugin.

### Metagenomic reads processing and annotation

For the shotgun metagenomic raw reads, quality was checked with FastQC v0.11.8 (Andrews 2010). Nextera adaptors were removed with Trim Galore v0.5.0 (Krueger 2012). Subsequently, low-quality sequences (Q < 20), indeterminated (N) bases, reads <50 nt, and 20 nt from 5′ end and 4 nt from 3′ end were removed with Cutadapt v1.15 (Martin 2011). Processed reads were assembled into contigs using MEGAHIT v1.1.3 (Li et al. 2015) with meta-large mode (a parameter specifically implemented to assemble complex metagenomes). The quality of assembly was evaluated with QUAST v5.0.2 metaQUAST mode (Mikheenko et al. 2016). Protein-coding genes were predicted with Prodigal v2.6.3 metagenomic mode (Hyatt et al. 2010). Metabolic and biogeochemical functional traits were predicted with METABOLIC-G v4.0 (Zhou et al. 2022). For the taxonomic annotation, the amino acid sequences were aligned against the Genome Taxonomy Database (GTDB) r95 protein set (Parks et al. 2021) with the BLASTp functionality of Diamond v2.0.5.143 (Buchfink et al. 2021), using a maximum e-value of 10^−7^, a query cover >30%, and a maximum number of target sequences per query of 4. To quantify the annotated genes, transcripts per million (TPM; counts per million for this article) were calculated with CoverM v0.6.1 contig mode (Woodcroft et al. 2021), considering a minimum percentage of identity of 95% and a minimum read alignment percentage of 50%. Statistical differences between the two metagenomes were assessed with the Statistical Analysis of Metagenomic Profiles (STAMP) v2.1.3 software (Parks et al. 2014), using a two-sided Fischer's exact test with the Newcombe–Wilson confidence intervals calculation method (nominal coverage of 95%) and Storey false rate discovery multiple test correction (reported *q-*values) as recommended by the authors. Features with a difference between proportions effect size < 1.00, ratio of proportions effect size < 2.00, and *q*-values > 0.05 were not further considered.

### Statistical analysis

The physicochemical and amplicon processed data were exported to R v4.2.2 (R Core Team 2022) for further filtering and statistical analysis. Physicochemical data were standardized and then visualized using a Principal Components Analysis (PCA) to determine the most relevant environmental variables. The 16S rRNA and ITS singleton and doubleton reads were removed, as well as taxa with read counts <10^−5^. Variance analyses were performed for the physicochemical and amplicon data. The normality and homoscedasticity of the data were assessed using the Shapiro–Wilks and Levene tests, respectively. Both the *t*-test and the non-parametric Mann–Whitney Wilcoxon test with a Benjamini–Hochberg post-hoc correction, depending on the normality test results, were used to identify significant pairs. Analysis of Variance (ANOVA) and non-parametric Kruskal–Wallis tests were used to assess the global significance.

Beta-diversity metrics were calculated and manipulated using the *phyloseq* and *microbiome* R packages (McMurdie and Holmes 2013, Lahti and Shetty 2017). Sample-dissimilarity matrices were generated using the weighted Unifrac distance metric on log_10_-transformed data. Community structure was analyzed using Multidimensional Scaling (MDS) analysis. Permutational Analysis of Variance (PERMANOVA) was used to identify significant differences in community structure between sites and soil physicochemical conditions using the *vegan* R package (Oksanen et al. 2020). The variables evaluated were removed consecutively until all remaining variables were significant. Beta dispersion tests were performed to ascertain if the observed differences were influenced by dispersion. The drivers of the edaphic communities’ assembly were inferred by running the phylogenetic bin-based null model (iCAMP) with a randomization time of 1000, bin size limit of 48, and a threshold of phylogenetic distance of 0.2 (Ning et al. 2020).

## Results

### Soil physicochemistry and microbial community diversity

The soils from the arid Namaqua (NNP) and hyperarid Richtersveld (RNP) drylands showed very similar physicochemistries (Fig. S1 and Table S1). Both were oligotrophic and mainly composed of sand (59.4% ± 1.24 in Namaqua and 60.4% ± 1.73 in Richtersveld), followed by silt (33.6% ± 3.00 and 34.3% ± 2.30), and clay (7.0% ± 3.67 and 5.3% ± 0.98). Samples showed similar pH values, ranging between 6.2 and 7.4 (Fig. S1 and Table S1).

Five soils (NNP3A-D and NNP2C) from the arid Namaqua dryland presented significantly higher (Mann–Whitney Wilcoxon test: *p*-value < 0.05) ions (especially K^+^, Mg^2+^, and Na^+^) and nutrients (carbon, nitrate, and ammonium) concentrations, as well as clay content and electrical conductivity (Figs S1 and S2). A PCA ordination plot confirmed that ions and nutrient contents were important factors separating these saline soils from all the others, essentially along the PC1 axis which explained 61.6% of the samples’ variance (Fig. S3).

**Figure 2.**
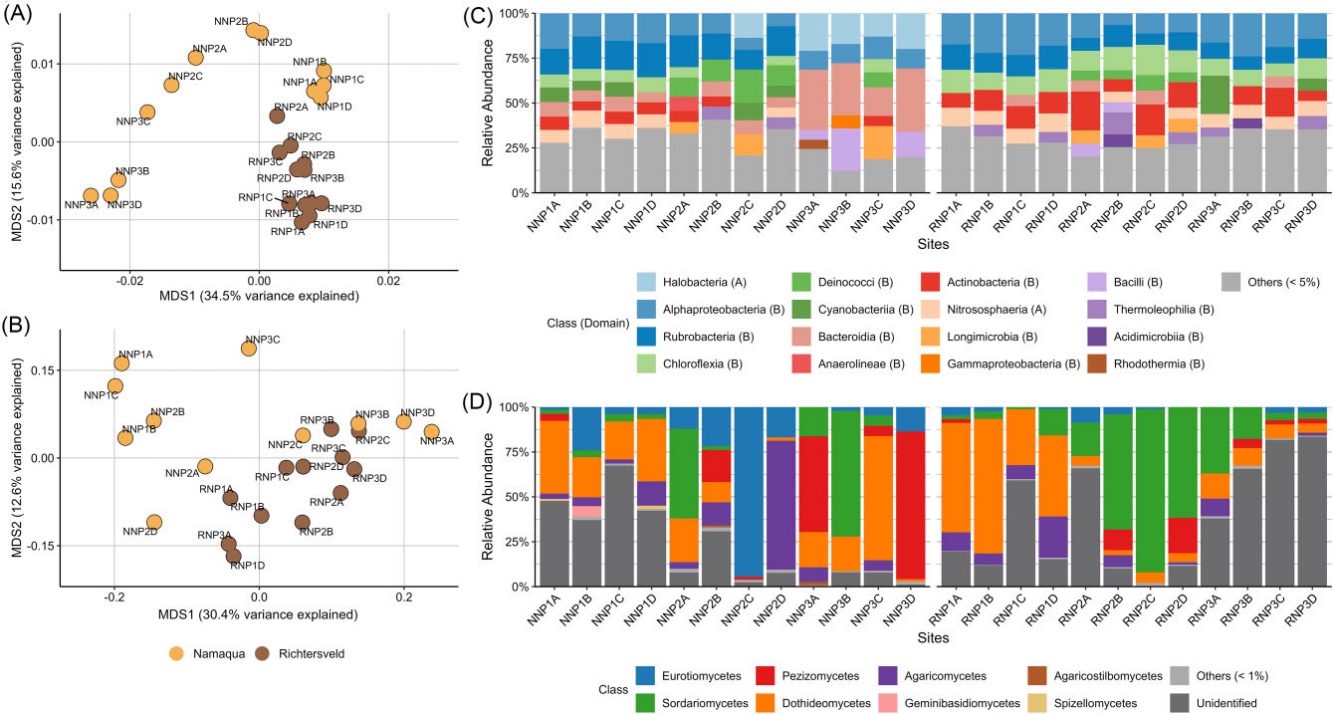
Southern African dryland edaphic microbial community diversity. MDS plots of bacterial and archaeal (A), and fungal (B) communities with weighted Unifrac distances calculated over log_10_-transformed data. The points are colored according to the dryland (NNP, Namaqua; RNP, Richtersveld), and the labels indicate the corresponding sample. Stacked bar charts showing class-level bacterial and archaeal (C), and fungal (D) composition with the respective domain in parentheses. A, Archaea; B, Bacteria.

The edaphic bacterial and archaeal (Fig. 2A), as well as fungal (Fig. 2B) communities’ beta-diversity, were clearly influenced by the aridity regime and soil salinity. When categorized into arid non-saline (NNP1A-D and NNP2ABD), saline (NNP3A-D and NNP2C), and hyperarid (all RNP samples), the bacterial and archaeal communities were significantly grouped by salinity (PERMANOVA: arid vs. saline, *F* = 5.87, *p*-value = 6.00e^−3^; hyperarid vs. saline, *F* = 11.5, *p*-value = 6.00e^−3^; Table S2), but not aridity (arid vs. hyperarid comparison, *F* = 2.26, *p*-value = 0.07). In contrast, both aridity and salinity significantly impacted fungal communities (arid vs. saline comparison, *F* = 2.66, *p*-value = 0.01; hyperarid vs. saline, *F* = 2.54, *p*-value = 0.02; and arid vs. hyperarid, *F* = 4.03, *p*-value = 6.00e^−3^; Table S2). Other factors significantly affecting the beta-diversity of the Bacteria and Archaea communities were Mg^2+^ and Ca^2+^ concentrations, while ion (K^+^, Na^+^, and Ca^2+^) and nutrient (ammonium and nitrate) concentrations, carbon content, and pH significantly influenced the beta-diversity of fungal communities (Table S2).

Members from the Bacteria domain dominated all the Bacteria and Archaea communities, with abundances of 90.0% ± 5.8 and 92.8% ± 2.2 in the arid Namaqua and hyperarid Richtersveld, respectively (Fig. 2C). The most represented phylum globally was Actinomycetota, with an average relative abundance of 20.4% ± 12.0 and 31.5% ± 4.2 in the Namaqua and the Richtersveld soils, respectively, followed by Pseudomonadota (14.7% ± 4.1 and 17.4% ± 5.4), as described in other drylands (Leung et al. 2020). The most abundant classes were Rubrobacteria (phylum Actinomycetota; 16.7% ± 1.8 and 10.2% ± 2.5 in the Namaqua and in the Richtersveld soils, respectively), Alphaproteobacteria (Pseudomonadota; 13.7% ± 4.1; 16.4% ± 5.4), Actinobacteria [or Actinomycetia (Parte et al. 2020), phylum Actinomycetota; 6.0 ± 1.1; 11.7% ± 5.0], and Chloroflexia (Chloroflexota; 6.3% ± 1.4; 11.3% ± 2.5). The radiotolerant bacterial genus *Rubrobacter* (Actinomycetota; 10.9% ± 7.8 and 10.2% ± 2.5) and the archaeal class Nitrososphaeria (Thermoproteota phylum) were also found abundant in the bacterial and archaeal communities (6.7% ± 2.0 and 7.2% ± 2.2; Fig. 2C and Table S3). We also noted that the saline NNP arid soils were particularly enriched in members of the Bacteroidia (Bacteroidota; 24.4% ± 11.9) and Bacilli (Bacillota; 9.5% ± 9.4) classes and members of the halophilic archaeal class Halobacteria (Halobacteriota; 16.9% ± 3.6), belonging to the *Halorussus, Natronomonas, Candidatus* Halobonum, and *Haladaptatus* genera (Fig. 2C and Table S3).

Dryland edaphic fungal communities were dominated by members of the phylum Ascomycota (66.2% ± 28.1 in Namaqua and 56.1% ± 28.4 in Richtersveld), followed by Basidiomycota (13.8% ± 20.9 and 8.7% ± 7.3; Table S3). However, compared to Bacteria and Archaea, the fungal communities were more variable in nature; i.e. they were found to be more sampling site- and dryland-specific. For example, the fungal community from Namaqua site 1 (NNP1) was enriched in members of Dothideomycetes class (29.7% ± 9.5), especially the genus *Kalmusia* (7.3% ± 2.2 of the site community), while site NNP2 was enriched with members of Eurotiomycetes class (36.3% ± 39.0), particularly *Knufia* (13.9% ± 4.2; Fig. 2D and Table S3). Similarly, while the site RNP1 community was dominated by members of Dothideomycetes class (53.1% ± 19.0), especially *Curvularia* (37.4% ± 11.2), the site RNP2 community was dominated by members of Sordariomycetes class (58.9 ± 30.0), particularly *Monosporascus* (58.7% ± 29.9; Fig. 2D and Table S3). Our results also showed that most of the dryland fungal ASVs could not be assigned to a taxonomic clade, confirming that dryland fungal communities remain substantially undercharacterized (van der Walt et al. 2016, Cowan et al. 2020, Vikram et al. 2023).

### Edaphic dryland microbial communities’ assembly under poly-extreme conditions

**Figure 3.**
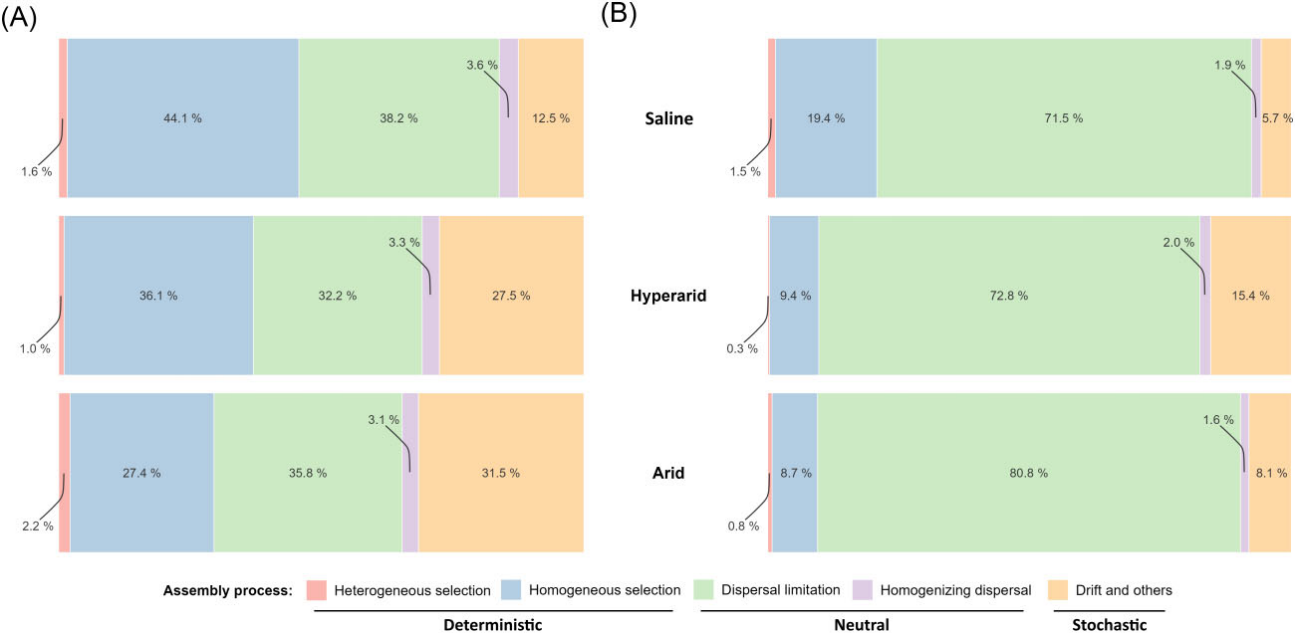
Ecological processes driving Southern Africa dryland soil microbial communities assembly. Stacked bar chart showing the contribution of each process to the assembly of the hyperarid, arid, and arid saline soil bacterial and archaeal (A), and fungal (B) communities.

Using the iCAMP framework (Ning et al. 2020), we evaluated the relative contributions of deterministic, neutral and stochastic processes on the assembly of the arid non-saline, arid saline and hyperarid bacterial and archaeal, and fungal communities (Fig. 3). Independently of the soil properties, Bacteria and Archaea community assembly was most influenced by three mechanisms, namely deterministic homogeneous selection (27.4%–44.1%), neutral dispersal limitation (32.2%–38.2%), and stochasticity (12.5%–31.5%; Fig. 3A). Furthermore, within the hyperarid Richtersveld and the saline Namaqua sites, the deterministic homogeneous selection was significantly higher than for the arid non-saline Namaqua sites (Cohen's d: hyperarid vs. arid comparison, effect size = 4.39, *p*-value = 1.68e^−3^; arid vs. saline comparison, effect size = –2.46, *p*-value = 0.01; Table S4). Fungal community assembly was mostly influenced by neutral dispersal limitation (71.5%–80.8%; Fig. 3B). We note that, even if not significantly different, homogeneous selection was the highest for the arid saline soil fungal community (19.4% vs. 9.4% and 8.7%).

### Functional profiling of the arid saline (Namaqua) and hyperarid (Richtersveld) soil communities

To better understand how edaphic microbial communities adapt to poly-extreme environmental selective pressures, we analyzed shotgun metagenomes from the arid saline NNP3 Namaqua and the hyperarid RNP3 Richtersveld sites (Figs 1 and S1, and Table S1).

#### Stress response genes diversity and abundance

**Figure 4.**
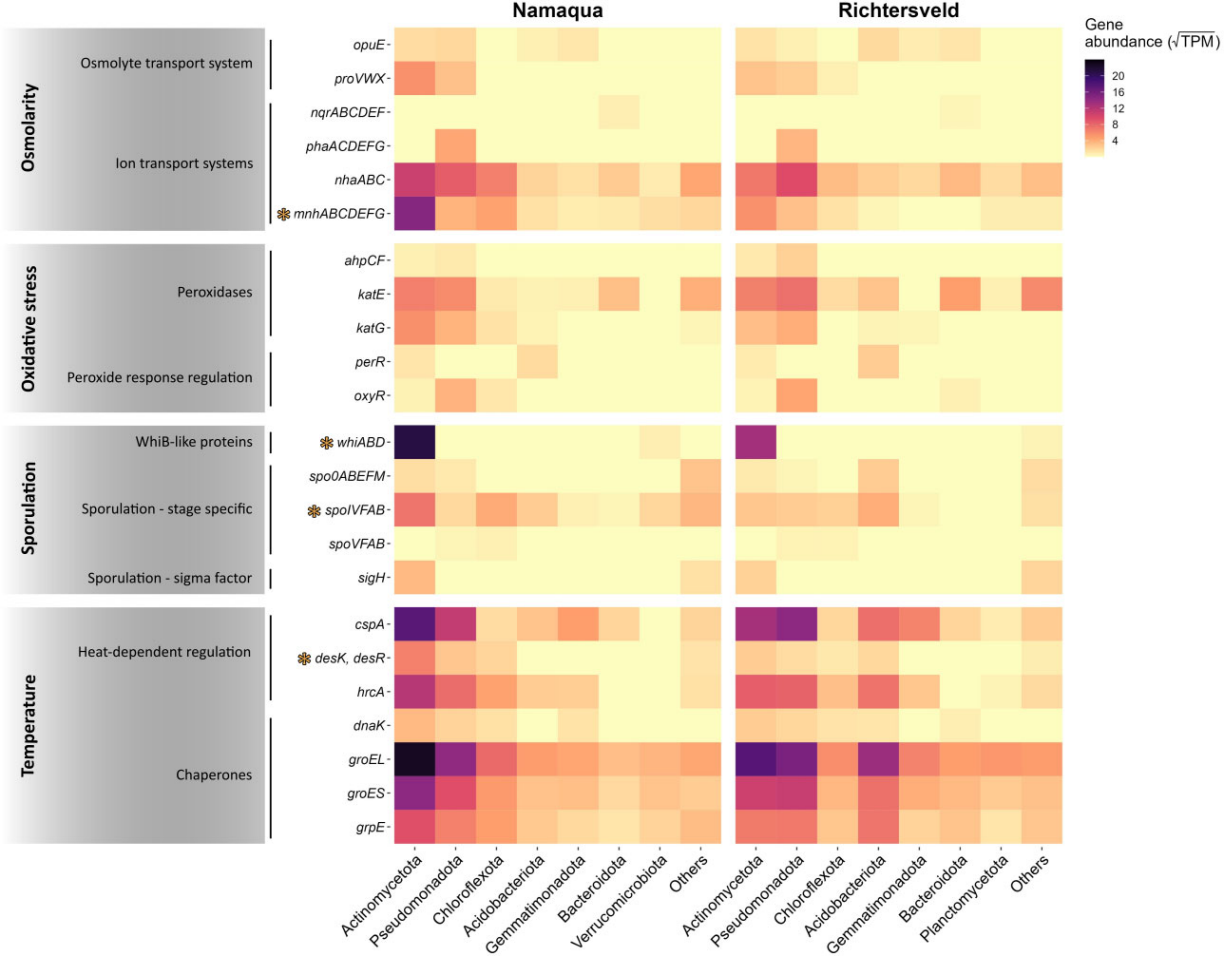
Abundance and distribution of key functional genes related to microbial stress response in Southern African dryland soils. The seven phyla with the highest gene abundances (>35.2 TPM) in both drylands are shown in the *x*-axis, whilst less represented phyla are grouped in the “Others” category. Colored asterisks indicate the gene families that were significantly enriched in one of the drylands (two-sided Fischer's exact test; *q*-value < 0.05). The TPM values are squared-root transformed.

To evaluate the potential adaptation strategies of dryland microbial communities to the poly-extreme conditions, we first evaluated the presence and abundance of genes related to osmotic regulation, sporulation, heat shock, and oxidative stress response. Both communities displayed similar stress response gene profiles (Fig. 4). The canonical bacterial heat shock protein family D (Hspd60) member 1 [or *groEL* (Hayer-Hartl et al. 2016)] was the most abundant gene in both metagenomes and showed similar abundances in both metagenomes (888.96 TPM in the hyperarid Richtersveld metagenome and 871.22 TPM in the arid saline Namaqua dataset; Table S5). Similarly, the highly abundant chaperonin cofactor *groES* and the CspA cold shock protein genes (*groES*: 348.85 TPM in Richtersveld, 359.13 TPM in Namaqua; *cspA*: 488.51 and 480.04 TPM) showed similar abundances in both metagenomes. Interestingly, both the GroEL–GroES complex and the CspA protein genes were associated with many different bacterial phyla, being most abundant in Actinomycetota, Pseudomonadota, and Acidobacteriota (Fig. 4). Similarly, most of the osmolyte and ion transporters genes, such as the *nha*ABC (Na^+^/H^+^ antiporter), *pha*ACDEFG (K^+^/H^+^ antiporter), or *pro*VWX and *opu*E (osmolytes transport systems), did not significantly varied between communities (Fig. 4 and Table S5). The *nha* genes were also widespread in the communities, while only members of the Pseudomonadota (especially of the genus *Microvirga*) contained the *pha* genes.

Four out of 23 of the stress response gene families (i.e. 17.4%) detected were significantly enriched in the Namaqua arid saline metagenome (two-sided Fischer's exact test: *q*-values < 0.05; Figs 4 and S4): the *mnh*ABCDEFG genes of Na^+^/H^+^ antiporter, the Des pathway *des*K and *des*R genes [involved in the thermal control of membrane unsaturated fatty acids to ameliorate the effects of temperature changes (Aguilar et al. 2001)], and the *spoIVF*AB and *whi*ABD genes [both associated with sporulation (Dong and Cutting 2003, Bush 2018)]. All but the *whi*ABD genes (only encoded by members of the Actinomycetota phylum) were assigned to multiple phyla. Overall, these results suggest that both communities have similar stress response capacities, but that the communities in saline systems require additional mechanisms to survive under high osmotic stress conditions.

#### Potential nutrient cycling capacities

**Figure 5.**
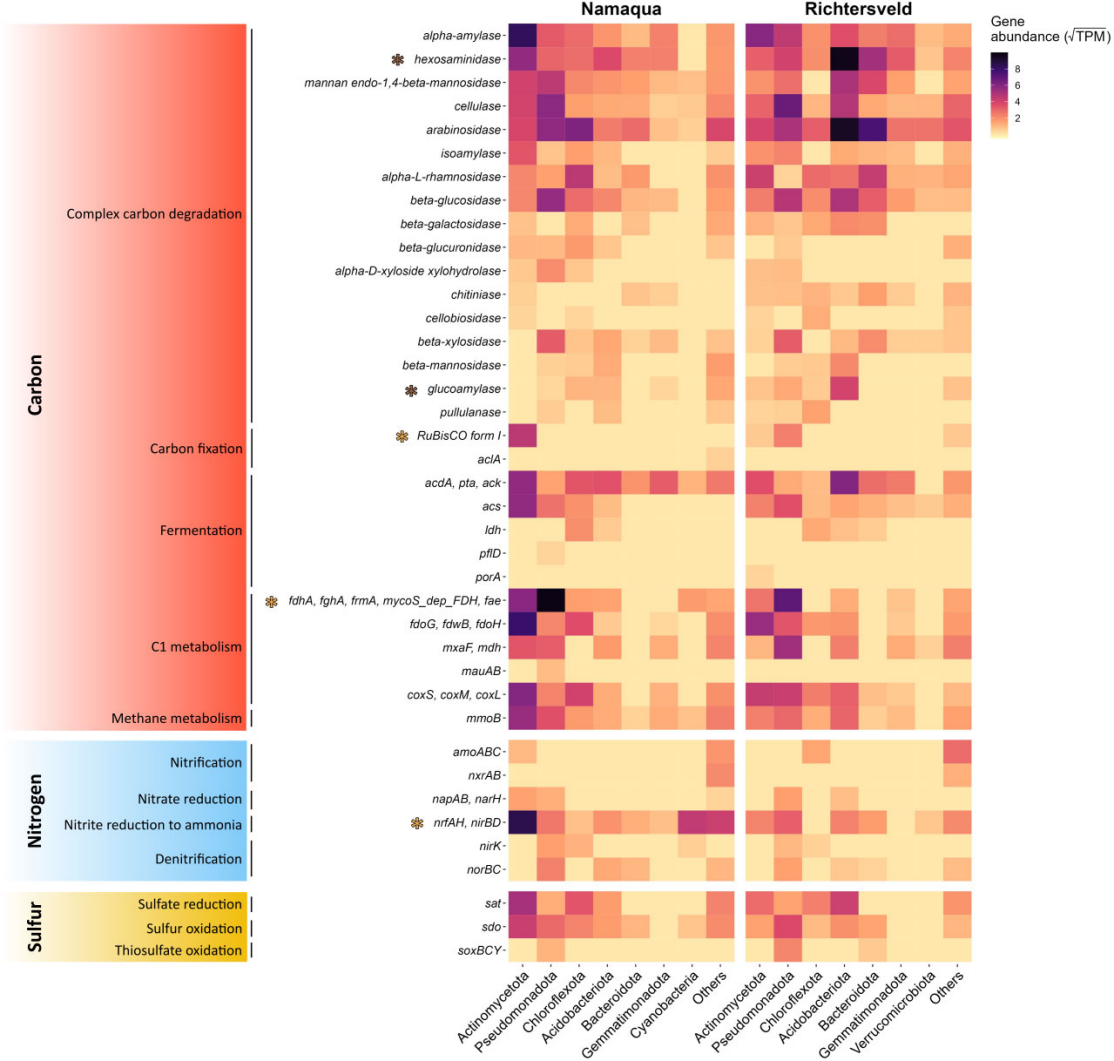
Abundance and distribution of key functional genes related to microbial metabolism and nutrient cycling in Southern African dryland soils. Genes related to carbon, nitrogen, and sulfur cycling pathways are displayed. The seven phyla with the highest gene abundances (>13.5 TPM) in both drylands are shown in the *x*-axis. The less represented phyla are grouped in the ‘Others’ category. Colored asterisks indicate the gene groups that were significantly enriched in one of the drylands (two-sided Fischer's exact test; *q*-value < 0.05). The TPM values are squared-root transformed. mycoS_dep_FDH: mycothiol-dependent formaldehyde dehydrogenase.

The potential nutrient cycling capacities of the edaphic communities were evaluated using key metabolic marker genes from carbon, nitrogen, and sulfur cycling pathways (Fig. 5). As observed for the stress-related genes, their abundances were generally similar in both metagenomes. This was particularly evident for some of the most abundant and widespread genes in both metagenomes, such as those related to the degradation of complex carbon molecules (e.g. alpha-amylase; with 101.70 and 88.90 TPM in Namaqua and Richtersveld metagenomes, respectively), C1 metabolism, particularly formate (*fdo*G, *fdw*B, and *fdo*H; 86.55 and 52.35 TPM) and methanol (*mxa*F and *mdh*; 32.22 and 43.26 TPM) oxidation genes, and acetogenesis (*acd*A*, ack*, and *pta*; 80.80 and 70.20 TPM) and acetate metabolism (*acs*; with 44.71 and 25.92 TPM) fermentation genes (Fig. 5). Only three gene groups related to organotrophic metabolism varied significantly between the two metagenomes (Fig. S4): hexosaminidase and glucoamylase carbohydrate-active enzymes (CAZymes) genes were significantly more abundant (two-sided Fischer's exact test: *q* = 2.98e^−9^ for the hexosaminidase, *q* = 0.01 for the glucoamylase) in the hyperarid Richtersveld samples compared to the arid saline Namaqua samples (169.17 and 19.56 TPM vs. 78.43 and 4.79 TPM, respectively), while the formaldehyde metabolism genes (*fdh*A, *fgh*A, *frm*A, mycoS_dep_FDH, and *fae*) were significantly more abundant (*q* = 1.11e^−7^) in the Namaqua metagenome (86.55 and 52.35 TPM, respectively; Figs 5 and S4). Overall, this suggests that the two edaphic communities have a similar high potential capacity for organotrophic growth using available soil organic matter.

The capacity for carbon fixation in dryland soil communities appeared to be limited and restricted to only a few microbial taxa (Fig. 5). Of the six known carbon fixation pathways (Fuchs 2011), only the Calvin-Benson-Bassham (CBB) and the reductive tricarboxylic acid (rTCA) cycles were detected. The CBB RuBisCO form I gene was detected in both dryland sites, being significantly more abundant (*q* = 0.035) in the Namaqua (21.49 TPM) metagenome than in the Richtersveld dataset (6.78 TPM; Figs 5 and S4). This gene was assigned mainly to *Gaiella occulta* (class Thermoleophilia, phylum Actinomycetota) and members of the genus *Pseudonocardia* (Actinomycetia) in the arid saline Namaqua, and members of the genus *Bradyrhizobium* (Alphaproteobacteria) in the hyperarid Richtersveld. The rTCA ATP-citrate lyase *acl*A gene was observed only in trace quantities in the Namaqua metagenome (0.13 TPM).

No significant differences in abundance were identified between dryland sites for the chemolithotrophic oxidation-related genes (Fig. 5). The presence of the nitrification *amo*ABC (NH_3_ → NH_2_OH) and nitrite oxidation *nxr*AB genes, and glutathione persulfide (GSSH) *sdo* and thiosulfate (S_2_O_3_^2−^) *sox*BCY oxidation genes suggest that both communities have similar potential capacities to oxidize nitrogen and sulfur species for energy purposes (Simon and Klotz 2013, Wu et al. 2021). Other sulfur cycling genes such as sulfide (S^2−^) oxidation *fcc*B and *sqr* genes, reported in other drylands (Jordaan et al. 2020, Ortiz et al. 2021), were not detected in our study.

As atmospheric trace gas harvesting and metabolism have recently been found to be a key adaptative feature of dryland microbial communities (Greening and Grinter 2022), we evaluated the presence of key metabolic marker genes in our metagenomes (Fig. 5). Atmospheric CO oxidation genes (*cox*S, *cox*M, and *cox*L) were detected in both metagenomes and found to be widespread across different taxa, and particularly within members of the genera *Geodermatophilus* and *Blastococcus* (class Actinomycetia), *Microvirga* (Alphaproteobacteria), and *Rubrobacter* (Rubrobacteria; Table S5). We also detected the methane oxidation gene *mmo*B (soluble methane monooxygenase), especially associated with members of the genus *Rubrobacter* (class Rubrobacteria) and *Desertimonas* (Acidimicrobiia) in Namaqua soils, and with members of the class Blastocatellia (phylum Acidobacteriota) in Richtersveld soils. H_2_ oxidation [Ni-Fe]-hydrogenase group 1 genes were only detected in the Namaqua metagenome (3.87 TPM) and were only encoded by *Conexibacter woesei* (Actinomycetota, class Thermoleophilia) and *Luteimonas* sp. (Pseudomonadota, class Gammaproteobacteria; Table S5). These results suggest that the Namaqua and Richtersveld microbial communities could potentially obtain energy from the oxidation of atmospheric CO, and to a lesser extent H_2_, as observed in drylands worldwide (Jordaan et al. 2020, Bay et al. 2021).

Reductive pathways were also detected in similar abundances in both metagenomes (Fig. 5). The denitrification Cu-containing nitrite reductase *nir*K (NO_2_^-^ → NO) and nitric oxide reductase *nor*BC (NO → N_2_O) genes were detected in both drylands (Fig. 5). However, the nitrous oxide reductase *nos*DZ genes (N_2_O → N_2_) and the cytochrome *c* nitrite reductase *nir*S were not observed. This suggests that the Namaqua and Richtersveld communities might not be able to generate N_2_ as a final denitrification product. Only the nitrite reduction to ammonia (NO_2_^−^ → NH_4_^+^) genes *nrf*AH and *nir*BD showed a significant difference (*q* = 1.96e^−14^; Fig. S4), being more abundant in the Namaqua metagenome than in the Richtersveld dataset (125.18 TPM and 29.03 TPM, respectively; Table S5). The sulfate (SO_4_^2−^) reduction *sat* gene was the only reductive pathway from the sulfur cycle detected in both metagenomes.

#### Evidence of functional redundancy in poly-extreme edaphic habitats

Metabolic functions can be typically performed by multiple coexisting, taxonomically distinct microorganisms, a phenomenon that is known as functional redundancy (Louca et al. 2018). Our results clearly showed that members of different taxonomic groups were potentially capable of performing similar ecosystem functions (Fig. 5). This implies a high level of functional redundancy in dryland microbial communities. This was especially true for genes related to complex carbon degradation, as 14 of the 17 genes identified (82.25%) were assigned to more than five different phyla. C1 metabolism gene sets such as the *fdh*A, *fgh*A, *frm*A, mycoS_dep_FDH, and *fae* were potentially encoded by 60 and 61 genera in Namaqua and Richtersveld samples, respectively. Such redundancy was also observed for carbon monoxide oxidation (*cox*S, *cox*M, and *cox*L, with 74 and 54 encoding genera), methane oxidation (*mmo*B, with 73 and 56 encoding genera), nitrite reduction to ammonia (*nrf*AH*, nir*BD, with 65 and 56 encoding genera), sulfur oxidation (*sdo*, with 43 and 37 encoding genera), and sulfate reduction (*sat*, with 32 and 45 encoding genera) genes.

## Discussion

The vegetation of NNP and the RNP are described as part of the Succulent Karoo and Desert biomes of South Africa (Mucina and Rutherford 2006). NNP occurs in the Succulent Karoo Biome, with a winter rainfall of <200 mm per year (arid) as well as the common fog, also called by the local Nama communities the *Malmokkie*, coming from the Atlantic Ocean during the mornings (Juergens et al. 2013). The RNP occurs in two biomes, namely the Succulent Karoo and Desert. The Succulent Karoo Biome in the RNP is associated with the mountain ranges of the park, while the Desert Biome is associated with the inland of the RNP (Fig. 1). The rainfall of the Desert Biome is <40 mm per year (hyperarid) and most of the time received summer rain associated with relatively short (20–30 min) thunderstorms which sometimes could take years to fall (Mucina and Rutherford 2006, Juergens et al. 2013).

### Namaqua and Richtersveld soils contain typical dryland microbial communities

The Namaqua and Richtersveld edaphic bacterial communities exhibited compositions similar to those described for other dryland soils worldwide (León-Sobrino et al. 2019, Bay et al. 2021, Meier et al. 2021, Cowan et al. 2022, Marasco et al. 2022a), with most reads affiliated to members of the bacterial Rubrobacteria, Alphaproteobacteria, Actinobacteria, and Chloroflexia classes. The genus *Rubrobacter*, a highly radiation and desiccation-resistant taxon (Rao et al. 2016, Meier et al. 2021), which was particularly dominant in each Southern African dryland was also found a prevalent member of Colorado Plateau (Osman et al. 2021), Atacama Desert (Crits-Christoph et al. 2013, Schulze-Makuch et al. 2018), Namib Desert (Naidoo et al. 2022), eastern Australia (Delgado-Baquerizo et al. 2020) and central Tibet edaphic communities (Rao et al. 2016). Its successful colonization of dryland soils may be related to its mixotrophic lifestyle (Meier et al. 2021), given its potential capacity to metabolize C1 compounds (formate and methanol), ferment (acetogenesis and acetate metabolism), degrade complex carbohydrates (e.g. arabinoside and hexosamine), and oxidize atmospheric traces gases (CO and CH_4_).

The chemolithoauthotrophic ammonia-oxidizing Nitrososphaeria [phylum Thermoproteota, formerly Thaumarchaeota (Rinke et al. 2021)] was the most abundant member of the arid non-saline Namaqua soils and hyperarid Richtersveld archaeal communities, as observed in other drylands (Makhalanyane et al. 2015, Vikram et al. 2016, Ren et al. 2018, Huang et al. 2019). Multiple nitrification *amo*ABC gene sequences were affiliated with members of this class, suggesting that this taxon drives the accumulation of nitrate in Southern Africa (and other) dryland soils (Delgado-Baquerizo et al. 2016).

Similarly, the Southern African dryland soil fungal communities were dominated by members of Ascomycota (Dothideomycetes [notably the genus *Curvularia*], Sordariomycetes [*Monosporascus*], and Pezizomycetes), as already observed (Makhalanyane et al. 2015, van der Walt et al. 2016, Murgia et al. 2019, Cowan et al. 2020, Marasco et al. 2022b, Vikram et al. 2023, 2023). However, a significant proportion of their members could not be assigned more specifically, which indicates that fungal communities are deserving of more extensive investigation in drylands worldwide (Gómez-Silva et al. 2019, Murgia et al. 2019).

### Salinity and aridity shape Southern African dryland soil microbial communities

Climatic and environmental factors can directly (e.g. soil physicochemistry) and indirectly (e.g. by altering the plant coverage) influence edaphic communities (Maestre et al. 2015, Fierer 2017, de Vries et al. 2018, Cui et al. 2019, Huang et al. 2019, Gao et al. 2021, Hu et al. 2021). Our results show that salinity, but not aridity, significantly influenced Namaqua and Richtersveld edaphic Bacteria and Archaea community composition and diversity, in a process related to niche partitioning (Stomeo et al. 2013, Johnson et al. 2017, Huang et al. 2019, Marasco et al. 2022b). Higher salinity led to a decrease in microbial diversity, and an increase in the importance of deterministic processes (i.e. environmental filtering) and community dissimilarity (Zhang et al. 2019). The observation that the saline soils studied were enriched in halophilic archaea (Halobacteria) is consistent with the findings of other saline dryland soils such as the Atacama (Crits-Christoph et al. 2016, Schulze-Makuch et al. 2018, Gómez-Silva et al. 2019), the Namib (León-Sobrino et al. 2019), and the Tarim Basin (Ren et al. 2018, Yang et al. 2022) deserts.

A phylogenetic bin-based null model analysis showed that deterministic homogeneous selection mechanisms were significantly more important for the Bacteria and Archaea communities from the most extreme sites, i.e. the arid saline Namaqua and hyperarid Richtersveld. The stronger selective pressures imposed on their indigenous Bacteria and Archaea communities could favor processes such as environmental filtering, selecting only the most adapted members, and therefore changing the community structure according to the environmental conditions (Louca et al. 2018, Huang et al. 2019, Song et al. 2019, Li et al. 2023).

Fungal communities showed a less obvious site- and dryland-specific heterogeneity. Although aridity and salinity influence their beta-diversity, high compositional variability was evident for all sites. The assembly results showed that dispersal limitation mechanisms were the main contributor to the edaphic fungal community assembly, suggesting that ecological barriers affect their dispersal (Vikram et al. 2023). As previously described (Zhou and Ning 2017), low dispersal rates resulting from dispersal limitation could increase community turnover, and therefore promote the differentiation of communities between sites. It is well established that the versatility, sporulation capacity, and hyphae morphology of fungi contribute to their high tolerance to poly-extreme conditions, possibly reducing the influence of abiotic stress variables (aridity and salinity) on community composition (Sterflinger et al. 2012, Murgia et al. 2019, Remke et al. 2021, Coleine et al. 2022).

### Functional redundancy buffers the effect of structure changes on communities’ functionality

Although the hyperarid Richtersveld soils were clearly less vegetated than the arid saline Namaqua sites (Fig. 1), both communities showed a similar abundance in genes related to carbon cycling; and particularly highly abundant CAZymes genes, which catalyze the breakdown, synthesis, and modification of carbohydrates such as those found in plant matter (López-Mondéjar et al. 2016). This suggests that both communities have the necessary genetic capacity to take advantage of the abundant plant biomass produced scant after rain events (Armstrong et al. 2016). Although we detected CBB and rTCA carbon fixation genes, their abundances were low compared to other pathways, and their distribution was limited to a few bacterial taxa. While autotrophic carbon fixation has been shown to potentially occur (Vikram et al. 2016, Ren et al. 2018, Gómez-Silva et al. 2019, León-Sobrino et al. 2019, Jordaan et al. 2020, Bay et al. 2021), with significant CO_2_ fixation rates, particularly in less vegetated dryland soils (Chen et al. 2021, Zhao et al. 2018), our findings suggest that this process likely plays a much smaller role compared to heterotrophic carbon recycling.

The oxidation of atmospheric trace gases provides an important continuous-energy-harvesting strategy for dryland soil microorganisms (Jordaan et al. 2020, Leung et al. 2020, Bay et al. 2021, Ortiz et al. 2021). While hydrogenase genes were only detected in Namaqua soils, carbon monoxide dehydrogenase, and methane monooxygenase genes were abundant and widespread in Namaqua and Richtersveld communities. Methanotrophy has only been described for members of the Pseudomonadota (Alpha and Gammaproteobacteria), Verrucomicrobia, Methylomirabilota, and Halobacteriota phyla (Guerrero-Cruz et al. 2021). In our results, most of the methane monooxygenase gene reads were assigned to members of the Acidobacteriota and Actinomycetota phyla, which potentially broadens the spectrum of methane oxidizers. Overall, these results suggest that Southern African dryland microbial communities are well adapted to meet their energetic demands during the frequent starvation periods (i.e. limited water and organic carbon) by energy acquisition by gas harvesting (Leung et al. 2020, Greening and Grinter 2022). Interestingly, metabolic pathways such as carbohydrate and hydrogen oxidation are also water-producing processes (e.g. 2H_2_ + O_2_ = 2H_2_O), potentially supplementing the water requirements of desiccated cells (Cowan et al. 2023).

Nitrogen is frequently considered the second most limiting resource in dryland environments after water (Pointing and Belnap 2012). Dinitrogen fixation (also known as diazotrophy) is the most important pathway for nitrogen acquisition in many ecosystems (Ramond et al. 2022). However, the cleavage of the N_2_ triple bond requires large amounts of energy and water, neither of which is abundant in dryland soils (Ramond et al. 2022). The absence of diazotrophy markers (i.e. *anfDKG, nifHDK*, or *vnfDKG*) in our two metagenomes confirms that this process is largely absent in superficial soil ecosystems (Ren et al. 2018, León-Sobrino et al. 2019). Dryland superficial and sub-superficial soils are in generally very rich in biologically assimilable nitrate (NO_3_^−^) (Walvoord et al. 2003, Graham et al. 2008). The high prevalence of genes encoding for the reduction of nitrate (NO_3_^−^) to nitrite (NO_2_^−^), and the subsequent reduction to ammonia (NH_4_^+^), demonstrate that the Namaqua and Richtersveld microbial communities take advantage of the nitrate/nitrite soil pools as an alternative strategy to obtain bioavailable nitrogen and storage energy (Gómez-Silva et al. 2019, León-Sobrino et al. 2019, Ramond et al. 2022). The particularly high concentrations of nitrate in the saline Namaqua soils would explain the significantly higher abundance of nitrate reduction genes in this dryland metagenome.

The detection of Sox system genes in the metagenomes is suggestive of a potential oxidative activity of chemolitoautotrophs on S^0^, thiosulfate, and sulfite pools (Vikram et al. 2016, Wu et al. 2021) resulting from the weathering of gypsum (CaSO_4_⋅2H_2_O; especially abundant in saline dryland systems) (Eckardt and Spiro 1999, Cowan et al. 2020, Voigt et al. 2020) or the Fe^3+^-dependent oxidation of metal sulfides (e.g. pyrite, FeS_2_) (Schippers 2004, Vera et al. 2022). As for reductive pathways, even though the ATP sulfurylase *sat* gene (first step of the sulfate reduction) was detected, the absence of other “signature” genetic markers for the reduction of sulfate (SO_4_^2−^) to sulfite (SO_3_^2−^) such as the *apr*A gene, and the subsequent reduction to sulfide (S^2−^) such as the dissimilatory sulfite reductase (Dsr) *dsr*AB or *asr*ABC genes (Wu et al. 2021) suggests that anaerobic sulfate reduction metabolism is mostly limited [but not exclusively; see (Peters and Conrad 1995)] to anoxic environments such as waterlogged soils where SO_4_^2−^ and not O_2_ has to be used as an electron acceptor (Pester et al. 2012, Štovíček et al. 2017). Previous meta-omics studies from the Atacama and Namib deserts also reported the absence of this pathway (Vikram et al. 2016, Gómez-Silva et al. 2019).

Since microorganisms must accumulate ions or compatible organic solutes to maintain a high intracellular osmotic pressure in highly saline environments (Saccò et al. 2021), we expected to see a higher abundance of genes related to osmolyte and ion transporters genes, generally widespread and abundant in dryland (Fierer et al. 2012, León-Sobrino et al. 2019, Song et al. 2019, Yang et al. 2022) and hypersaline (Martínez-Alvarez et al. 2023) environment microbiomes, in the saline soil metagenome. However, only the *mnh*ABCDEFG Na^+^/H^+^ antiporter genes were significantly enriched in the saline soil metagenome, while other salt resistance genes (e.g. those encoding glycine betaine and proline, and Na^+^ and K^+^ transporters) were equally abundant in both metagenomes. Similarly, genes implicated in resistance to thermal shock, particularly the GroEL–GroES complex that prevents protein aggregation under heat-shock conditions (Llorca et al. 1998), and the CpsA cold-shock protein (Jiang et al. 1997), showed similar abundances in the two communities studied. The presence of these thermal shock genes is a signature of microbial adaptation to arid environments (Schulze-Makuch et al. 2018, León-Sobrino et al. 2019), and could be associated with the extreme diurnal and seasonal temperature variations common in drylands (Pointing and Belnap 2012, Gunnigle et al. 2017), with the Richtersveld NP and the Namaqua NP presenting mean monthly maxima and minima of 44.9°C and 30°C and 1.9°C and 8°C, respectively (Mucina and Rutherford 2006), and to the simultaneous impacts of osmotic and oxidative stresses (Keto-Timonen et al. 2016). Altogether, our results strongly suggest that both communities have developed similar adaptation mechanisms to thrive in these highly saline and temperature-variable environments.

Even though the microbial community structures from both drylands were differentially influenced by aridity and salinity, their potential stress response and nutrient cycling capacities were similar. Functional redundancy has been identified as the most important feature determining the functional robustness of soil microbial communities (Eng and Borenstein 2018). In our study, the apparent functional redundancy at phylum level, particularly within genetically diverse phyla such as Actinomycetota and Pseudomonadota (Barka et al. 2016, Kirchberger et al. 2020), could compensate for the differences in community composition (Louca et al. 2018), where loss of taxa might be functionally compensated by the presence of different taxa capable of performing the same function (Philippot et al. 2013, Louca et al. 2018), as observed in multiple habitats including dryland soils (Nelson et al. 2016). Such process should underpin the resilience, functional stability, and long-term adaptation of such communities to environmental perturbations (Louca et al. 2018, Biggs et al. 2020), and suggests that the Namaqua and Richtersveld soil communities may survive under the more stressful environmental conditions predicted for the ongoing climate change.

## Final remarks

Changes in local soil (e.g. soil physicochemical) or climatic conditions are known to significantly alter microbial community compositions and functional status in dryland soils (Fierer et al. 2012, Magalhães et al. 2014, Scola et al. 2018, Song et al. 2019, Li et al. 2022). Here we demonstrated that soil salinity and hyperaridity significantly influenced the beta-diversity and taxonomic composition of dryland edaphic bacterial, archaeal, and fungal communities. However, only the bacterial and archaeal community assembly was influenced by these poly-extreme conditions, increasing the relevance of deterministic homogeneous selection in a process linked to niche partitioning. We particularly noted the similarities in functional capacities and stress adaptation strategies in these communities. We suggest that the high levels of functional redundancy implied from analyses of pathway diversity may buffer the effects of community structure variation on the potential community functionality. However, due to the complex interactions between the microorganisms and the environment and between different trophic tiers in the community, and the inherent limitations of metagenomics analyses (e.g. it is not possible to know if the genes detected are being expressed, or if they come from live or dead cells), future research should incorporate multi-omics and experimental biochemical approaches to understand the actual functions that are being carried out by soil communities *in situ* (i.e. the metaphenome) (Quince et al. 2017, Jansson and Hofmockel 2018). Overall, our findings suggest that soil ecosystems in Southern African drylands could potentially remain functionally stable, and that dryland edaphic microbial communities may retain functionality even under more severe conditions of aridity and salinity, as might be expected with the trajectories of global aridification and salinization driven by climate change.

## Supplementary Material

fiae157_Supplemental_Files

## Data Availability

The sequencing data is available in the NCBI Sequence Read Archive (SRA) under BioProject accession number PRJNA1067640 for paired-end reads (joined reads) for 16S rRNA and ITS genes amplicon sequencing and raw paired-end reads for the shotgun metagenomes.
