# Supplementary material for: Functional redundancy buffers the effect of poly-extreme environmental conditions on southern African dryland soil microbial communities
Source: FEMS Microbiol Ecol. 2024 Nov 20;100(12):fiae157. doi: 10.1093/femsec/fiae157 (PMC11636270; doi:10.1093/femsec/fiae157)
Supplement: fiae157_Supplemental_Files [file fiae157_supplemental_files.zip › Data_availability_access.pdf]

The sequencing data is available in the NCBI Sequence Read Archive (SRA) under BioProject accession number PRJNA1067640. The BioProject and associated SRA metadata (release date: 2024-12-27) are available as read-only format at:

https://dataview.ncbi.nlm.nih.gov/object/PRJNA1067640?reviewer=vqujmdq7crrsbfl8p2ensfr8uu
