# Supplementary material for: Functional redundancy buffers the effect of poly-extreme environmental conditions on southern African dryland soil microbial communities
Source: FEMS Microbiol Ecol. 2024 Nov 20;100(12):fiae157. doi: 10.1093/femsec/fiae157 (PMC11636270; doi:10.1093/femsec/fiae157)
Supplement: fiae157_Supplemental_Files [file fiae157_supplemental_files.zip › Tables_S2_S4_supplementary_data.docx]

**Table S2. Differences in microbial community beta-diversity matrix according to a PERMANOVA including climatic and environmental properties.**

| **Independent variables** | **R^2^** | **F** | **p-value** |
| --- | --- | --- | --- |
| **Bacterial and archaeal communities** | | | |
| Condition * | 0.07 | 2.75 | 0.04 |
| Hyperarid vs arid | 0.12 | 2.26 | 0.07 |
| Hyperarid vs saline | 0.43 | 11.5 | 6.00e-3 |
| Arid vs saline | 0.37 | 5.87 | 6.00e-3 |
| Mg^2+^ (mg L^-1^) | 0.15 | 5.97 | 4.00e-4 |
| Ca^2+^ (mg L^-1^) | 0.07 | 2.85 | 0.04 |
| Residual | 0.46 |  |  |
| **Fungal communities** | | | |
| Condition * | 0.12 | 4.85 | 1.00e-4 |
| Hyperarid vs arid | 0.19 | 4.03 | 6.00e-3 |
| Hyperarid vs saline | 0.15 | 2.54 | 0.02 |
| Arid vs saline | 0.21 | 2.66 | 0.01 |
| K^+^ (mg L^-1^) | 0.15 | 6.07 | 1.00e-4 |
| Na^+^ (mg L^-1^) | 0.14 | 5.74 | 1.00e-4 |
| Ca^2+^ (mg L^-1^) | 0.08 | 2.99 | 4.00e-4 |
| %C | 0.09 | 3.49 | 4.00e-4 |
| Ammonium (mg L^-1^) | 0.08 | 2.98 | 2.40e-3 |
| pH | 0.07 | 2.85 | 4.30e-3 |
| Nitrate (mg L^-1^) | 0.06 | 2.19 | 0.02 |
| Residual | 0.38 |  |  |

* An independent pairwise PERMANOVA was performed to compared between groups.

**Table S4. Summary of effect size (Cohen’s d) and significance between groups for each community assembly process.**

|  | | **Heterogeneous selection** | | **Homogeneous selection** | | **Dispersal limitation** | | **Homogenizing dispersal** | | **Drift and others** | |
| --- | --- | --- | --- | --- | --- | --- | --- | --- | --- | --- | --- |
| **Group 1** | **Group 2** | **Effect size** | **p-value** | **Effect size** | **p-value** | **Effect size** | **p-value** | **Effect size** | **p-value** | **Effect size** | **p-value** |
| **Bacterial and archaeal communities** | | | | | | | | | | | |
| Hyperarid | Arid | -1.49 | 0.13 | 4.39 | **1.68e-3** | -0.59 | 0.27 | 0.96 | 0.22 | -1.18 | 0.17 |
| Hyperarid | Saline | -0.89 | 0.24 | -1.23 | 0.18 | -0.90 | 0.20 | -0.69 | 0.29 | 4.78 | **8.48e-3** |
| Arid | Saline | 0.55 | 0.36 | -2.46 | **0.01** | -0.49 | 0.30 | -1.72 | 0.11 | 4.32 | **2.25e-3** |
| **Fungal communities** | | | | | | | | | | | |
| Hyperarid | Arid | -1.47 | 0.14 | 0.47 | 0.40 | -2.16 | 0.05 | 0.43 | 0.49 | 3.00 | **0.01** |
| Hyperarid | Saline | -1.00 | 0.36 | -1.44 | 0.13 | 0.79 | 0.33 | 0.19 | 0.56 | 3.05 | **0.01** |
| Arid | Saline | -0.52 | 0.43 | -1.60 | 0.10 | 1.47 | 0.12 | -0.61 | 0.33 | 1.11 | 0.21 |
