# Supplementary figures and images for: Functional redundancy buffers the effect of poly-extreme environmental conditions on southern African dryland soil microbial communities
Source: FEMS Microbiol Ecol. 2024 Nov 20;100(12):fiae157. doi: 10.1093/femsec/fiae157 (PMC11636270; doi:10.1093/femsec/fiae157)

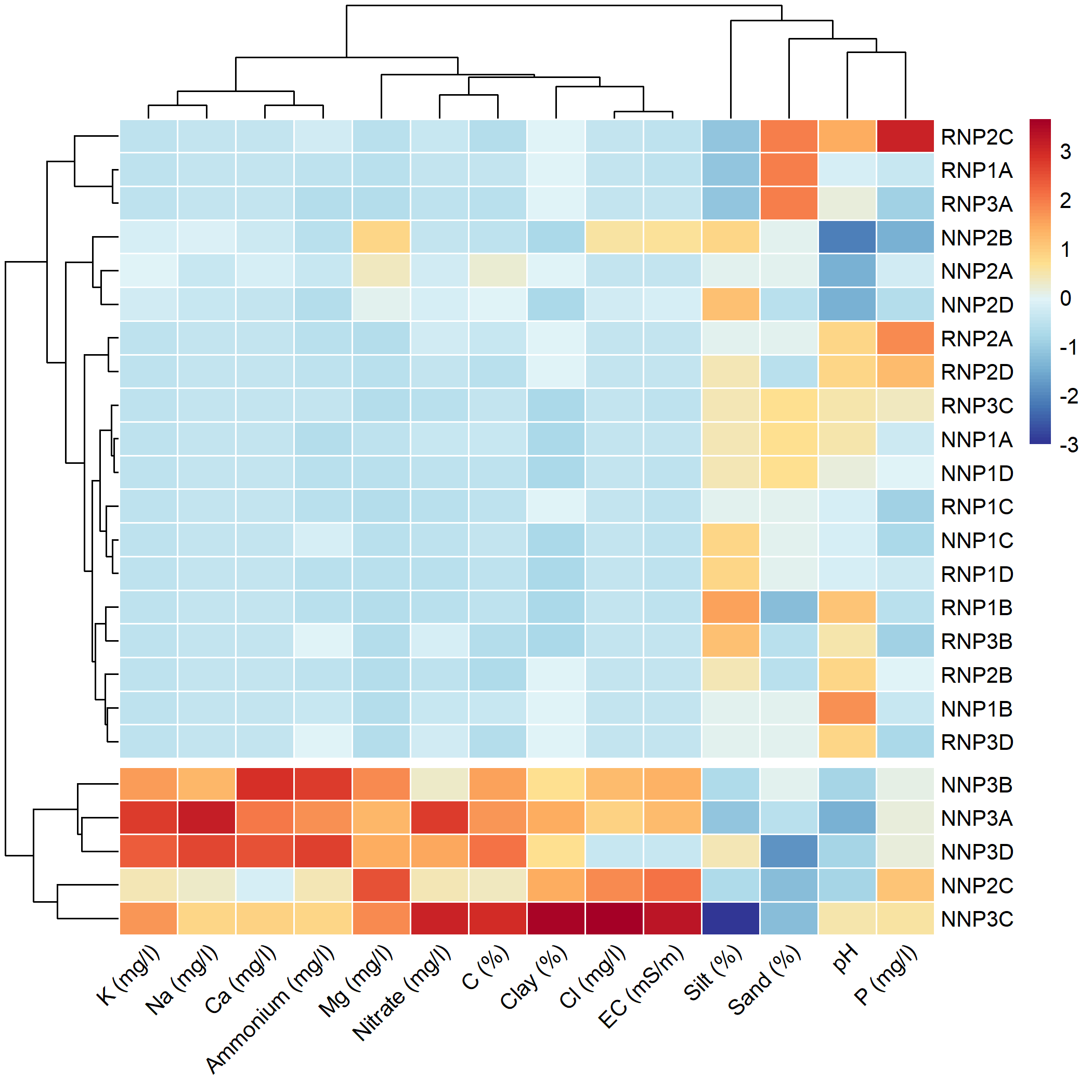

Supplement: fiae157_Supplemental_Files [file fiae157_supplemental_files.zip › Figure_S1_supplementary_data.png]

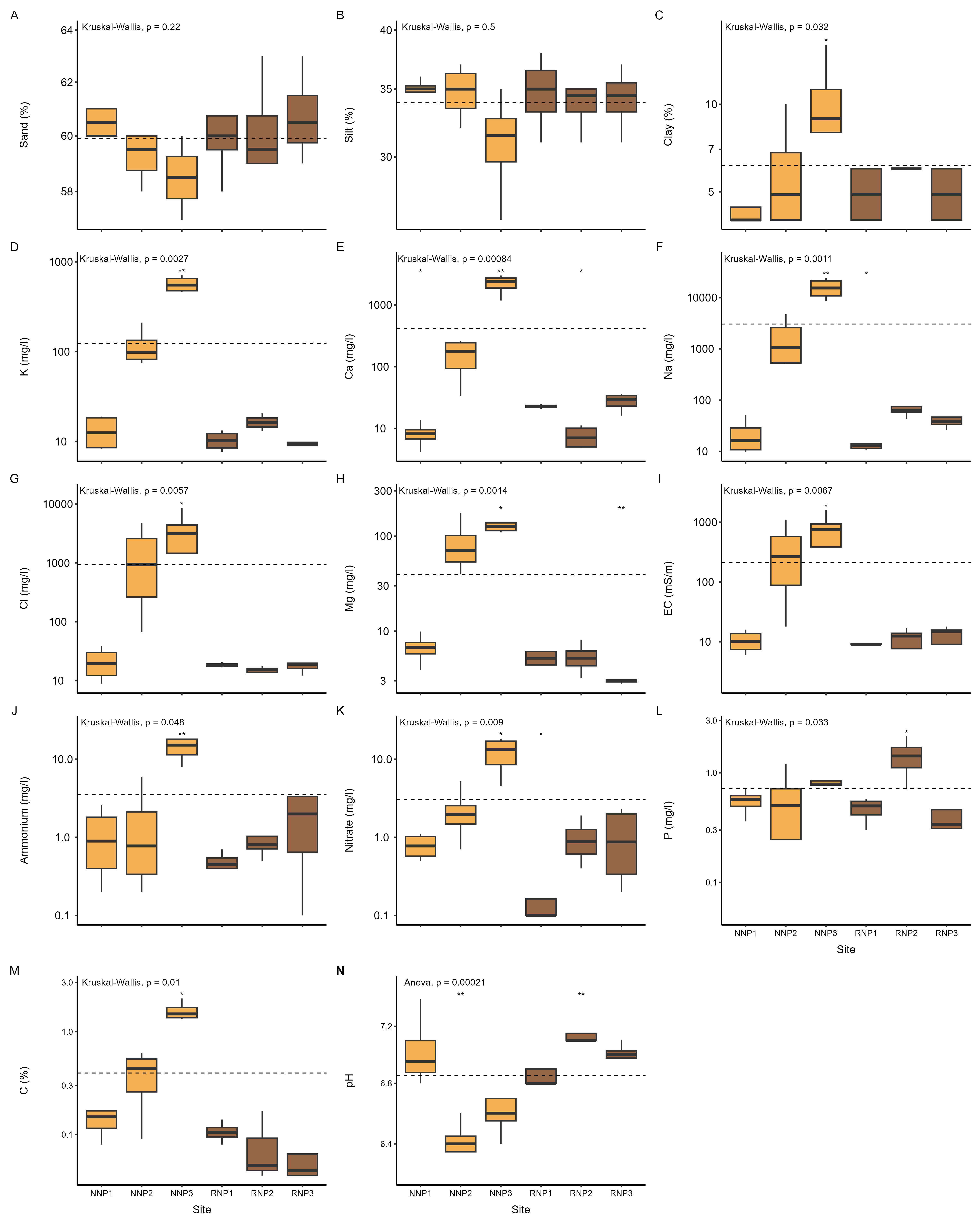

Supplement: fiae157_Supplemental_Files [file fiae157_supplemental_files.zip › Figure_S2_supplementary_data.png]

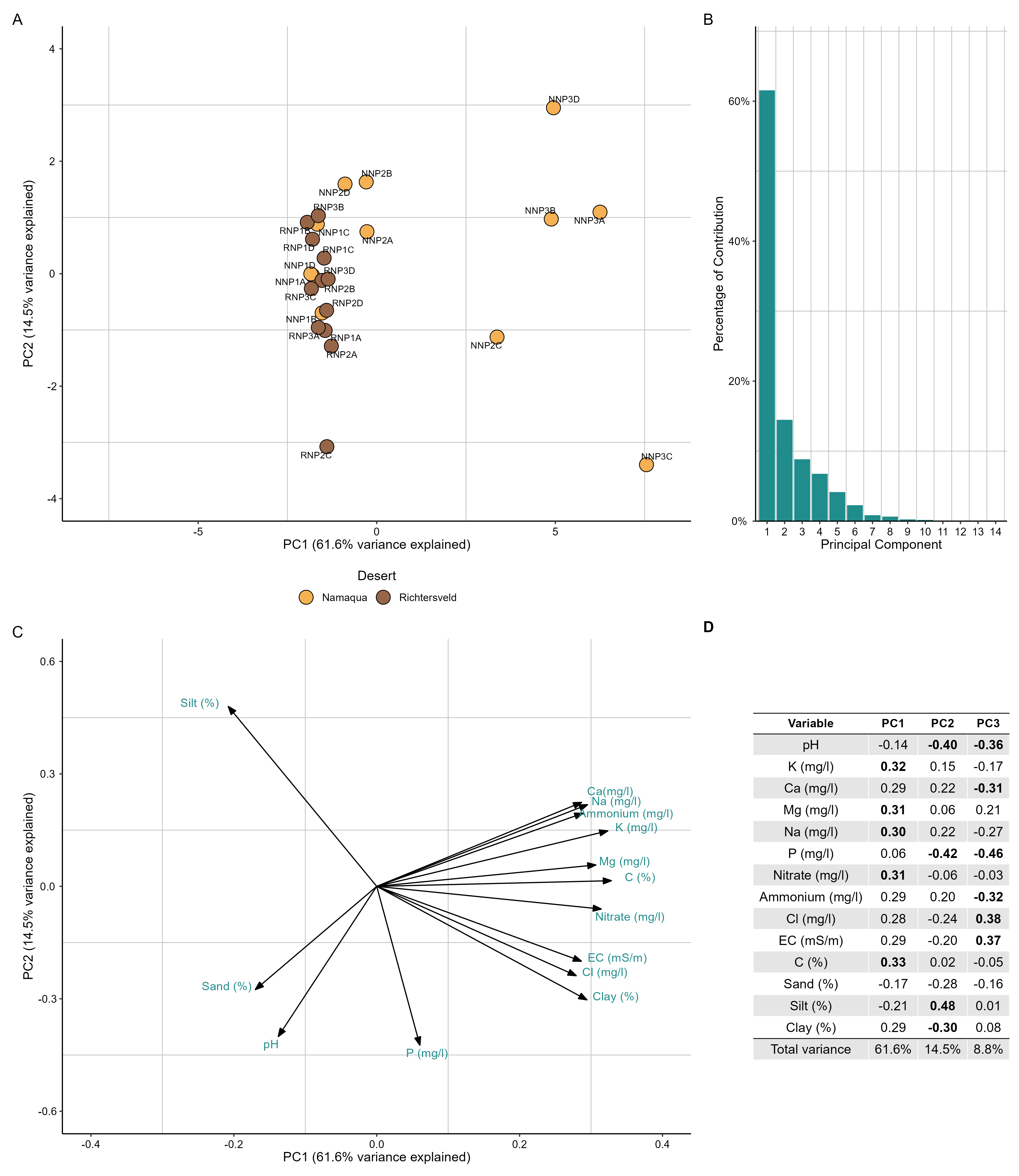

Supplement: fiae157_Supplemental_Files [file fiae157_supplemental_files.zip › Figure_S3_supplementary_data.png]

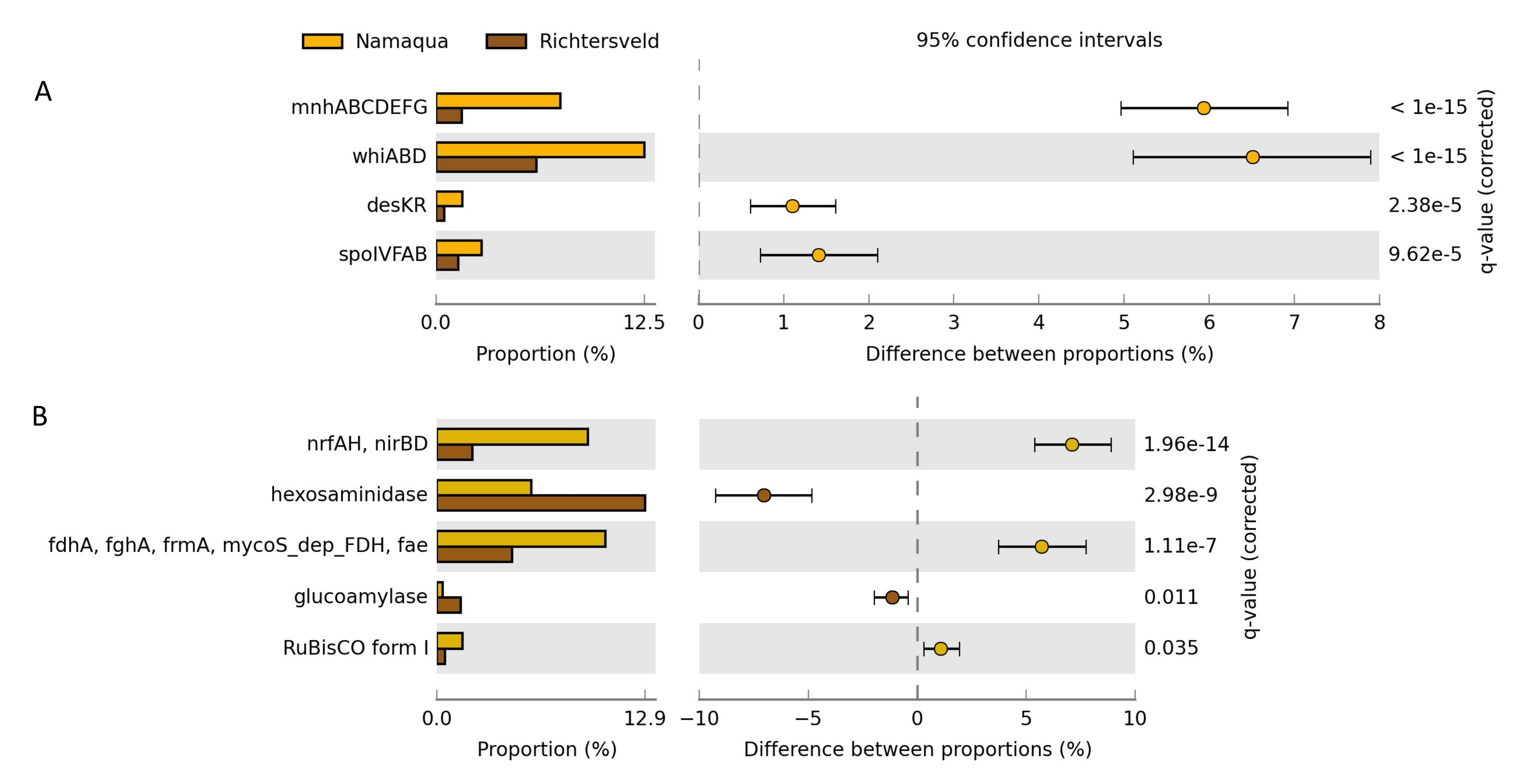

Supplement: fiae157_Supplemental_Files [file fiae157_supplemental_files.zip › Figure_S4_supplementary_data.png]
